# Doctors Disagree

**Published:** 1920-02-07

**Authors:** 


THE PUBLIC WELL-BEING—(*continued*)

### Doctors Disagree.

### Controversy over the Panel System.

THE contention of the Federation of Medical and Allied Societies that the public do not receive the best possible return for their expenditure under the Insurance Act is vigorously refuted by the medical secretary of the British Medical Association. No panel doctor, he says (in an interview published in the *Sunday Times*), would burke inquiry into the working of the system, but the present time is inopportune for a fair verdict. He recalls the suspicions amid which the system was started in 1913—resentment on the part of the doctors, political antagonism on the part of a large section of the public, and a general feeling that Mr. Lloyd George's alluring phrases were too good to be true. Then, just as the doctors were settling down to their work, the war intervened, and a depleted profession had to carry on as best it could. Therefore he advocates another year's normal trial before full investigation is held.

This seems a reasonable contention, and it is certainly a reasonable defence, so far as it goes, of the deficiencies of the system. But it scarcely seems conclusive against the case for immediate inquiry and remedies, and it is doubtful whether the public will agree with the secretary of the B.M.A. that inquiry shall not cover the question as to how the panel system has worked. This appears essential, not only on public health but also on political grounds. It is difficult to see how a system can be improved, unless a clear view is obtained as to how it has worked in the past.

The other point on which swords are crossed with the Federation has regard to the alleged inefficiency of the service. The secretary of the B.M.A. emphatically denies that the general practitioner is restricted to a few words and a bottle of not too expensive medicine.

"Roundly speaking (he says) there are 14,000 doctors who are doing this insurance work throughout the country, and I am sure that if you asked the average doctor in the average industrial district, he would tell you he is not conscious of labouring under any disabilities so far as his patients are concerned. Doctors have their grievances against some of the regulations, but these are mainly of a technical character, which do not particularly concern the public.

"But within the range of the service—and it must be remembered that it was never intended at the outset to be more than a general practitioner service—the doctor is not hampered in giving to patients what they ought to have.

"Any limitations in the service apply to all forms of private practice, so far as the poorer classes are concerned, and the Ministry and the profession in combination are doing their best to get these limitations removed.

"Many of us have recognised for a long time that the service is not complete. But the whole matter is under consideration, and as Dr. Addison said, it would have been attended to long ago but for the war."

At all events, what is prefectly plain is that Dr. Addison should at least give a guarantee that inquiry will definitely be held within a given time. This undertaking he has not yet given. Both the B.M.A. and Federation spokesmen show that it is necessary.

